# Changes in sleep with transcranial magnetic stimulation in adults with treatment resistant depression: Preliminary results from a naturalistic study

**DOI:** 10.1192/j.eurpsy.2021.415

**Published:** 2021-08-13

**Authors:** A.I. Sonmez, R. Kay, S. Schmids, C. Peterson, A. Herman, A. Widge, Z. Nahas, C.S. Albott

**Affiliations:** Psychiatry And Behavioral Sciences, University of Minnesota, Minneapolis, United States of America

**Keywords:** repetitive transcranial magnetic stimulation, treatment-resistant depression, hypersomnia, Insomnia

## Abstract

**Introduction:**

Sleep disturbance specifically insomnia, non-restorative sleep, and hypersomnia are common symptoms of major depressive disorder (MDD). As it alleviates major depressive disorder, transcranial magnetic stimulation (TMS) may improve associated sleep disturbances, and may also have inherent sedating or activating properties.

**Objectives:**

To examine the impact of TMS on sleep disturbances in adults with treatment resistant depression in a clinical setting, we retrospectively reviewed de-identified data from naturalistically-treated MDD patients undergoing an initial acute course of TMS therapy at St.Louis Park MinCEP Clinic.

**Methods:**

Adults with treatment-resistant depression received daily TMS treatments. 9-item Patient Health Questionnaire (PHQ-9) total scores were used to calculate % change at endpoint (relative to pretreatment baseline); response on both measures was defined as 50% reduction in scores, with remission defined as a final total score 4 on the PHQ-9. Insomnia was measured with a 3-item subscale of the Inventory of Depressive Symptomatology Self Report (IDS-SR). Hypersomnia was measured with a single IDS-SR item. Pairwise comparisons were performed using Student’s T-test. Categorical variables were compared using Fisher’s Exact test. Continuous outcome measures were tested with an analysis of covariance, using baseline PHQ-9 score as a fixed effect covariate.

**Results:**

TMS appears to have differential modulatory effects on insomnia and hypersomnia in adults with treatment resistant depression.

**Conclusions:**

These results may provide the basis for further investigation into therapeutic applications of TMS in addressing sleep disturbances in treatment-resistant depression. Measures that separate hypersomnia and insomnia should be implemented in future work addressing effects of TMS in treatment-resistant depression.

**Disclosure:**

No significant relationships.

